# An Essay on Food, &c.

**Published:** 1839-04

**Authors:** 


Art. XIII.—*An Essay on Food, in which the received Doctrine of Modern Physiologists respecting the Waste of the Body is exploded; the cause of Animal Heat is explained upon New Principles; the source whence Nitrogen is derived by Herbivorous Animals is established; General Rules for the Preservation of Health are laid down; and the wisdom of the Divine Economy in all is vindicated.* By W. Grisenthwaite, author of a Refutation of Paine's Age of Reason, &c. —*London,* 1838. 8vo. pp. 119.

The refuter of Paine's Age of Reason, and the author of this long title-page, presents one of the many examples in the present day of persons determined to vindicate the Creator according to their own views, as if the wisdom of the creation was dependent for proof on their particular fancies. We consider all this kind of writing as very unprofitable. The religious are not edified by it, and in the irreligious it does but raise a sneer. We have no doubt that Mr. Grisenthwaite, who is not a medical man, is a highly respectable and intelligent person. He dates his book at Beckenham Manor House, in Kent, and dedicates it to the Earl of Leicester, more distinguished and better known as Mr. Coke, of Norfolk; whose life, the author says, furnishes the best commentary upon the doctrine contained in it. We venture to assert that the said preface is written by more than one writer. At all events, some passages are well written, and some very ill expressed.

Mr. Grisenthwaite considers that the "almost sole use of food" is to preserve the temperature of the body. Except during growth or after injuries, he maintains that "there are no such operations in the animal economy as nutrition, assimilation, or reparation of waste." He devotes some pages to showing how many medical authorities have supported the grievously erroneous notion that the body requires nourishment and repair. He proceeds to show that a certain quantity of carbon is consumed by the lungs, which precise quantity is furnished by the food; more carbon being evolved whenever food is taken. The heat thus produced is just that, he maintains, which is lost by the body; and the sanguiferous apparatus brings back the blood to the heart, before it has lost any of its heat, so as to keep the temperature everywhere equal. This doctrine and its consequences are elaborately defended by arguments drawn from chemistry. There is such an air of candour and good faith about the writer that we must quote a page of his conclusions:

"If any professional man should read this essay, and feel any qualms about the conclusions drawn in it, I beg to request of him to satisfy himself upon the following particulars, before he passes judgment. Are eleven ounces and a half of carbon consumed in respiration during twenty-four hours? And do thirty-five ounces of bread, or about twenty-nine ounces of bread, and between twelve and fourteen ounces of meat, just furnish that quantity? Do the kidneys secrete three pounds of fluid a day, consisting of substances which exactly contain the nitrogen found in these twelve or fourteen ounces of meat? Will eleven ounces and a half of carbon, on combination with oxygen gas, impart four degrees of temperature per hour to the whole system, supposed to weigh one hundred and forty pounds? And does the body lose more heat than this in the same time? And can any other method be devised, by which an *equable* temperature could be so admirably preserved? Does not the energy of the body—within certain limits—evidently depend upon this temperature? How can I prove that the body wastes? What new arrangements are made out of the waste elements? How do they get into the circulation? and why, when there, since they are composed of the *same* elements as the fibres that remain, do they not go again to repair that waste? And why does waste take place more rapidly during digestion? Why does the lion waste four times as fast as man, even when he lives in a state of inactivity? If he can answer all these questions differently from those contained in this essay, he will not only destroy all my conclusions, but scatter to the winds the labours of the greatest men that ever adorned the annals of science. To me I hope he will deal with a gentle hand, seeing that I write for men of my own size; and have only been drawn into these speculations from a love of truth, and a desire to benefit—however slender my effort—the generation in which I live." (p. 114.)

One piece of wisdom, at all events, Mr. Grisenthwaite has learned: namely, to live sparingly. Allowing that about thirty ounces of bread, or substances composed of the same elements, and twelve ounces of meat a day, are sufficient for a man who weighs ten stone, and who breathes twenty times in a minute; he says his own consumption of food (he breathes but fourteen times in a minute), and that of his children, is not equal to *half* that quantity, and their health is excellent. He is himself quite unacquainted with sickness: twenty ounces of food are his maximum, and he can walk seven miles "on a winter's day, that shuts the forty-ounce eaters within doors." Of ale, wine, or spirits, he takes none; but he now and then suffers a little punishment if he takes more than twenty ounces of *fluid* (tea or coffee) in a day. We hope he will long live to enjoy such rare health, and to meditate on chemical physiology.

